# Acute dissociation and cardiac reactivity to script-driven imagery in trauma-related disorders

**DOI:** 10.3402/ejpt.v3i0.17419

**Published:** 2012-11-27

**Authors:** Martin Sack, Melanie Cillien, James W. Hopper

**Affiliations:** 1 Department of Psychosomatic Medicine and Psychotherapy, Klinikum rechts der Isar, Technische Universität München, Germany; 2 Harvard Medical School & Outpatient Addictions Services, Cambridge Health Alliance, Cambridge, MA, USA

**Keywords:** Dissociative disorders, PTSD psychophysiology, stress reactivity, script-driven imagery

## Abstract

**Background:**

Potential acute protective functions of dissociation include modulation of stress-induced psychophysiological arousal. This study was designed to explore whether acute dissociative reactions during a stress experiment would override the effects of reexperiencing.

**Methods:**

Psychophysiological reactions during exposure to script-driven trauma imagery were studied in relation to acute responses of reexperiencing and dissociative symptoms in 61 patients with histories of exposure to a variety of traumas. Acute symptomatic responses were assessed with the Responses to Script-Driven Imagery Scale (RSDI), and participants were divided into four groups by median splits of RSDI reexperiencing and dissociation subscale scores.

**Results:**

In a comparison of the high RSDI reexperiencing groups with low versus high acute dissociative symptoms, the high dissociators exhibited significantly lower heart rate (HR) during trauma script and a significantly smaller script-induced decrease in parasympathetic cardiac activity. HR reactivity to the trauma script was negatively correlated with acute dissociative symptom severity.

**Conclusions:**

Acute dissociative reactions are a potential moderator of response to experimental paradigms investigating psychologically traumatized populations. We therefore suggest that future research on psychophysiological stress reactions in traumatized samples should routinely assess for acute dissociative symptoms.

Dissociation can be viewed as a partially adaptive response to emotionally overwhelming distressing events (van der Kolk, van der Hart & Marmar, 1996). Potential acute protective functions of dissociation include modulation of information processing and stress-related bodily reactions (Lanius et al., 2010; Pole, 2007). Associated symptoms are alterations in perception such as depersonalization, derealization, and amnesia, as well as loss of psychomotor and sensory function.

Since the earliest finding of elevated HR in war combatants (Da Costa, 1871), numerous studies have investigated emotional processing in individuals suffering from different traumatic events by studying psychophysiological reactions during standardized stressors or recall of individualized trauma reminders. Yet typically one-third of individuals who meet diagnostic criteria for posttraumatic stress disorder (PTSD) do not show heightened physiological responsivity to trauma-related reminders (Orr, Metzger & Pitman, 2002). One possible explanation for such “non-responding” to trauma-related stimuli is that acute dissociative reactions lead to disengagement of emotional reactivity and associated overmodulation of autonomic arousal (Lanius et al., 2010).

This explanation is consistent with the recently supported hypothesis that central nervous system mechanisms underlying dissociation involve corticolimbic disconnection and inhibition of emotional processing (Sierra & Berrios, 1998). That is, initial neuroimaging studies revealed increased activation of brain regions implicated in regulating emotional responses in depersonalization disorder and a dissociative PTSD sample (Lanius et al., 2002; Pain, Bluhm & Lanius, 2009; Phillips et al., 2001).

Investigating the relationship between dissociative symptoms and autonomic activity, Griffin, Resick & Mechanic (1997) reported that rape victims with PTSD, who had experienced high levels of peritraumatic dissociation, subsequently exhibited less physiological reactivity while talking about the event, compared to those with low peritraumatic dissociation. While these results were not replicated in some subsequent studies assessing the effect of peritraumatic dissociation on autonomic arousal (Kaufman et al., 2002; Ladwig et al., 2002; Nixon, Bryant, Moulds, Felmingham & Mastrodomenico, 2005), others have reported diminished autonomic responses in individuals with state dissociative symptoms assessed by interview (Koopman et al., 2004; McDonagh-Coyle et al., 2001; Sierra et al., 2002), and in those exhibiting acute dissociative symptoms (Ebner-Priemer et al., 2005; Lanius et al., 2002; Pole et al., 2005).

Several reasons may account for the inconsistency of findings in these studies. First, a variety of different populations have been studied, including acute traumatized individuals without a DSM PTSD diagnosis (Griffin et al., 1997; Nixon et al., 2005; Pole et al., 2005) and patients with chronic PTSD or with borderline personality disorder secondary to childhood sexual abuse (Ebner-Priemer et al., 2005; Lanius et al., 2002). Second, different stress paradigms were utilized, such as autonomic assessment during relatively long (5–10 min) unstructured traumatic narratives (Griffin et al., 1997; Nixon et al., 2005; Pole et al., 2005), startle probes (Ebner-Priemer et al., 2005; Ladwig et al., 2002; Sierra et al., 2002) and individualized trauma scripts (Kaufman et al., 2002; Lanius et al., 2002). Additionally, studies correlating retrospectively assessed dissociative symptoms during a traumatic event with stress responses in a laboratory situation some weeks later (Griffin et al., 1997; Nixon et al., 2005), or even after years (Kaufman et al., 2002; Ladwig et al., 2002), apparently are more prone to show negative results than studies investigating the effect of acute dissociative stress reactions provoked by the laboratory stressor itself (Ebner-Priemer et al., 2005; Lanius et al., 2002; Pole et al., 2005).

One open question is, whether and if so, how reexperiencing and dissociative symptoms interact in the context of acute stress reactions and exposure to trauma-related stimuli. Reexperiencing and dissociation are not necessarily antagonistically related, and some authors have suggested a possible dominant influence of reexperiencing on autonomic reactions by activation of the adrenergic system and subsequent sympathetic arousal (Ladwig et al., 2002; Pole et al., 2005). If this were true, it would follow that only dissociative reactions of a certain severity would be sufficient to override the effects of reexperiencing. To investigate this issue experimentally, it is necessary to assess both reexperiencing and dissociative symptoms evoked by a laboratory stressor or symptom provocation method. For the script-driven imagery paradigm (Pitman, Orr, Forgue, De Jong & Claiborn, 1987), widely used in PTSD psychophysiology research (Orr et al., 2002), this has recently become an option (Hopper, Frewen, Sack, Lanius & van der Kolk, 2007a).

The present study investigates acute reexperiencing and dissociative symptoms during a reminder of a most stressful traumatic event in a population with a broad spectrum of trauma-related disorders including a high prevalence of dissociative disorders. We theorized that reexperiencing and dissociation would exert antagonistic influences on psychophysiological stress reactions to script-driven trauma imagery. Since reexperiencing was expected to have a dominant influence on psychophysiological arousal, participants with high-reexperiencing/low-dissociation reactions were compared to those with high-reexperiencing/high-dissociation reactions. We predicted that greater acute dissociative symptoms would be associated with less psychophysiological arousal, that is, lower HR and higher parasympathetic cardiac activity.

## Material and methods

### Participants

All participants were part of a study investigating the relationship between trauma-related symptoms and autonomic regulation during exposure to a trauma reminder. Participants were recruited from consecutive outpatients seeking treatment for trauma-related psychological problems. Inclusion criteria comprised reported memories of at least one experience meeting the traumatic event criterion for PTSD in ICD-10 (World Health Organization [WHO], 1992), age between 18 and 65, and sufficient German language knowledge to complete the questionnaires. Exclusion criteria were trauma exposure within the previous 3 months and acute psychotic symptoms. Written informed consent was obtained from all participants, and the Research Ethics Committee of Hannover Medical School approved the study.

From a total of 102 eligible patients, 14 declined psychophysiological assessment because they believed it would be too distressing, 8 reported partial trauma-related amnesia, 5 were on beta-blocker medications, and 14 could not be included for scheduling or organizational reasons. Data collected for the present study were from the remaining 61 participants, 47 (77%) of whom were women. Participants had experienced a variety of traumatic events, with 56% (34) reporting sexual victimization, 70% (43) non-sexual violence, and a majority (70% or 43%) repeated assaults during childhood. Female patients in the study reported significantly more often multiple traumatization (78% or 37% vs. 43% or 6%, χ2: 6.7, *p*=0.018) and suffered more often from PTSD (76% or 36% vs. 43% or 6%, χ2: 5.7, *p*=0.024). Sample demographic information is presented in Table 1.

**Table 1:** Sample characteristics

*N*	61
Age (years, mean±SD)	34.5±10.8
Sex	14 men, 47 women
<12 years of education	41 (67%)
Currently in a relationship	30 (49%)
Multiple traumatization	43 (70%)
Psychopharmacological medication	22 (36%)

A PTSD diagnosis was established for 69% (42) of participants with the SCID PTSD module, and there was a particularly high 40% (25) prevalence of dissociative disorders as assessed with the SCID-D interview (Steinberg, 1994). A total of 31% (19) of participants had both PTSD and a dissociative disorder. Among those with ICD-10 dissociative disorders, six showed depersonalization/derealization symptoms, four a loss of sensory or motor function, three dissociative amnesia, two dissociative fugue, one a dissociative identity disorder, and nine a dissociative disorder not otherwise specified. Among the 12 patients without PTSD or a dissociative disorder, the likelihood of other diagnoses was assessed with a checklist for diagnosis of ICD-10 mental disorders (Hiller, Zaudig & Mombour, 1995), which revealed that five patients were suffering from an anxiety disorder, two had a depressive disorder, three an adjustment disorder, and two a somatoform disorder.

Medication was reported as follows: antidepressants *N*=14 (23%), benzodiazepines *N*=5 (8%), mood stabilizer *N*=4 (7%), analgesic medication *N*=2 (3%), and neuroleptics *N*=2 (3%). Taking no psychopharmacological or analgesic medication was reported by *N*=33 (54%) of all patients.

### Instruments

The Responses to Script-Driven Imagery Scale (RSDI) (Hopper et al., 2007a) was developed to provide a brief and face valid measure of state PTSD and dissociative symptoms elicited by script-driven imagery, a widely used symptom provocation method in PTSD research. The 11-item RSDI provides an excellent factor structure for measuring state reexperiencing, avoidance, and dissociative symptoms evoked by script-driven trauma imagery. The predicted three-factor solution was strongly supported by confirmatory factor analyses, including tests for sample invariance across measurement and structural models in three different samples, with the fully constrained model exhibiting good model fit. The response format is a 7-point Likert scale, from 0 for “Not at all” to 6 for “A great deal,” with only those anchors at the extremes. The English RSDI was translated into German and back-translated to English. The last author (JWH) evaluated the English back-translated versions of the 11 final items as identical in wording or meaning to the original English language items. In the current study the RSDI was administered in questionnaire form, in the presence of the investigator to ensure comprehension of the directions and allow participants to ask for clarification about particular items. The RSDI has been found sensitive to functional neural response differences between reexperiencing and dissociation (Hopper, Frewen, van der Kolk & Lanius, 2007b).

The PTSD module (SCID PTSD) of the Structured Clinical Interview for DSM-IV Disorders (Spitzer, Williams, Gibbon & First, 1988) is a widely used semi-structured interview with good validity and reliability designed to assess the diagnosis of PTSD. We used the authorized German translation (Wittchen, Zaudig & Fydrich, 1997).

The Structured Clinical Interview for DSM-IV Dissociative Disorders (SCID-D) is a semi-structured interview with good reliability and validity designed to assess all five DSM-IV dissociative disorder diagnoses (Steinberg, 1994). The SCID-D has five numerical subscales and an overall score (SCID-D total), which was used in this study as an estimate measure of the overall severity of dissociative symptoms. We used the authorized German translation (Gast, Oswald, Zündorf & Hofmann, 2000).

The Impact of Event Scale (IES) is a 15-item self-report scale that is used to assess the frequency of intrusive and avoidant symptoms associated with the experience of a particular event (Horowitz, Wilner & Alvarez, 1979). Both the intrusion and avoidance scales have displayed an acceptable reliability (alpha of 0.79 and 0.82, respectively). Psychometric properties for the German translation have shown acceptable results (Ferring & Fillipp, 1994).

### Psychophysiological measurement

All trauma scripts were prepared by the study's principal investigator (MS), and described participants’ most disturbing traumatic events, sequentially unfolding the details in the present tense and first person. Scripts were then read to the patient to check for any inconsistencies with their memories, and recorded onto audiotape. The script-driven imagery procedure in this study differs from the standard approach (Pitman et al., 1987) by employing a script of 2 min rather than 30 sec in duration, and no imagery period after the script ends (Sack, Hopper & Lamprecht, 2004).

A second appointment was scheduled for the script-driven imagery sessions, approximately 1 week after script construction. The sessions took place in the therapy office that was best known to the patients to assure a sense of safety and familiarity within the surroundings. Participants were seated in a comfortable chair and asked to remain still during the recording procedure. After electrocardiogram (ECG) electrode placement and a 5-min adaptation period, a sequence of five scripts was played back via tape recorder, in a fixed order: (1) 2-min scripted relaxation exercise followed by a 1-min break; (2) 2-min neutral script of imagining washing dishes followed by 1-min break; (3) 2-min trauma script followed by a 5-min break; (4) repetition of scripted relaxation exercise and 1-min break; and (5) repetition of neutral script. Levels of subjective units of distress (SUDs) on an 11-point (0 to 10) scale were assessed immediately after the trauma script. The RSDI was completed immediately after the final neutral script to keep subjects focused on the script imaging procedure. SUD values were only assessed after trauma script for the same reason.

ECG signals were obtained via three commercial disposable Ag–AgCl electrodes placed on the chest and recorded in a miniaturized amplifier (Par-Port/F, Par-Elektronik, Berlin, Germany). Sampling rate of ECG data for acquisition of interbeat intervals (IBIs) was 1000 Hz. Finger pulse volume (FPV) was obtained via infrared plethysmography from a sensor attached to the index finger of the non-dominant hand with a Velcro belt. Changes in FPV served as an indicator of sympathetic arousal (Miller & Ditto, 1991). Respiration frequency was recorded from a piezoelectric sensor attached to the chest with an elastic chest belt and served as a control variable, since heart rate variability (HRV) is influenced by respiration. ECG, FPV, and respiration frequency were collected with the same instrument (Par-Port/F).

All psychophysiological data were transferred to a PC and a time series of interbeat intervals was generated. The time series of heart period data was visually displayed to edit outliers. Except for singular premature heart beats in two sessions, which were edited with a standard averaging procedure, all ECG data were free from artifacts and no further corrections were required.

For an estimation of basal parasympathetic cardiac activity, HRV was analyzed in the frequency band between 0.15 and 0.40 Hz (Berntson et al., 1997). For analysis of HRV in the time domain, the root mean square successive difference (RMSSD) was calculated for every heartbeat. According to published recommendations, the RMSSDs of five preceding and five following heart beats were specified (Task force of the European Society of Cardiology and the North American Society of Pacing and Electrophysiology, 1996). Due to the known skewed distribution of the RMSSD, the values were transformed with a natural logarithm for further statistical analyses.

Compared to frequency analysis (e.g., Fourier transformation), time series analysis of HRV using RMSSD is known to be of limited power in detecting intraindividual variations of parasympathetic tone (Berntson, Lozano & Chen, 2005). Despite this limitation, RMSSD was chosen as our estimate of parasympathetic drive, for two reasons: first, this measure is especially useful for detecting short-term variations. And second, by calculating RMSSD values for every heart beat, it is possible to graphically display changes in parasympathetic cardiac activity with an acceptable temporal resolution for depicting the time course of HR reactivity.

### Data reduction and statistical analyses

Mean values of all psychophysiological data were calculated for the first 60 sec of each script. Difference values were calculated by subtracting the average of the two neutral scripts from mean values during trauma script. HR data, FPV, and RMSSD were also averaged as segments of consecutive 5-sec intervals for graphical display. To investigate the association between script-related acute dissociative symptoms and psychophysiological arousal, Pearson correlation coefficients between psychophysiological data and script-related posttraumatic symptoms (i.e., RSDI subscale scores) were computed.

To test our hypothesis concerning differences in psychophysiological reactions during trauma script in participants with high vs. low acute dissociative symptoms, participants were divided by median split into high dissociating and low dissociating subjects. A second median split divided all participants into high-reexperiencing and low-reexperiencing groups resulting in four groups with nearly equal cell sizes. Reexperiencing was expected to exert a dominant influence on psychophysiological arousal. To detect influences of acute dissociative reactions, a comparison of the high-reexperiencing and low-dissociation response group (R+/D−) with the high-reexperiencing and high-dissociation response group (R+/D+) and a comparison between the low-reexperiencing and low-dissociation group (R−/D−) and the low reexperiencing and high-dissociation group (R−/D+) were carried out by analysis of variance (ANOVA) using the General Linear Model (GLM). For confirmation, ANOVA results were controlled by covariation analysis for: age, medication, gender, and respiration rate (RMSSD only). We predicted that comparison of the R+/D− and R+/D+ as well as R−/D− and R−/D+ would reveal significant differences in psychophysiological arousal, as indicated by post hoc pairwise comparisons. Significance levels were set at 0.05 for all statistical analyses, which were performed using the SPSS 12 statistical package (SPSS Inc., Chicago, IL, USA).

## Results

### Script-related subjective reactions

Most participants reported significant reexperiencing or avoidance symptoms in response to trauma script-driven imagery, with scores ranging between 0 and 6 (*M*=3.52, SD=1.63) and (*M*=3.73, SD=1.82), respectively. Scores for dissociation in response to trauma script-driven imagery ranged between 0 and 5.6 (*M*=1.82, SD=1.64). Subjective distress (SUD) during trauma script-driven imagery ranged between 2 and 10 (*M*=6.28; SD=2.24).

No significant inter-correlations between RSDI-subscales were found except for avoidance and dissociation (*r*=−0.27, *p*=0.035). SUD and RSDI-arousal were found to be significant positive correlated (*r*=0.60; *p*<0.001), whereas a significant negative correlation was observed between SUD and RSDI dissociation (*r*=−0.26, *p*=0.046).

### Script-related psychophysiological reactions

Regarding the entire sample, trauma script exposure led to a significant HR increase (80.5±11.6 vs. 89.9±15.7 bpm, *T*=6.9, *df*=60, *p*<0.001, *d*=1.8); a significant decrease of FPV (15.4%±9.4% vs. 10.0%±6.8%, *T*=7.3, *df*=60, *p*<0.001, *d*=1.9), indicating a significant increase of sympathetic activity and a significant decrease of parasympathetic cardiac activity as indexed by the logarithmically transformed RMSSD (3.33±0.64 vs. 3.17±70 ms, *T*=4.3, *df*=60, *p*<0.001, *d*=1.1). Respiration rate exhibited no significant script-related changes (16.0±3.9 vs. 16.4±4.3 bpm, *T*=1.1, *df*=60, *p*=0.30, *d*=0.28).

As indicated in Table 2, correlational analyses revealed significant medium to large positive correlations of RSDI reexperiencing with maximum HR during trauma script, HR change (trauma—neutral) and SUD during trauma script. RSDI-dissociation scores were found to have significant small and medium negative correlations with HR during trauma script and HR change, respectively. Acute dissociative symptoms also exhibited a significant medium-sized positive correlation with trait dissociative symptoms as measured by the SCID-D and a medium-sized negative correlation with age during first traumatization. SCID-D scores were significant negative correlated with age during first trauma (*r*=−0.54, *p*<0.001).

**Table 2:** Pearson correlations of script-related symptom responses with psychophysiological reactivity, symptom measures and age during first traumatization

	Responses to Script-Driven Imagery Scale		
	
Variables	Reexperiencing	Avoidance	Dissociation
HR maximum (bpm)	0.48***	−0.11	−0.30*
HR difference (bpm)	0.55***	−0.13	−0.26*
(ln)RMSSD difference (ms)	0.54***	−0.14	−0.21
FPV difference (%)	−0.21	−0.03	0.33**
SUD	0.60***	−0.16	−0.26*
IES total	0.26*	−0.30*	0.28*
SCID-D total score	0.10	−0.21	0.48***
Age during first trauma	0.17	0.04	−0.38**

HR: heart rate; RMSSD: root mean square of successive differences of interbeat intervals; FPV: finger pulse volume; SUD: subjective units of distress; IES: Impact of Event Scale; SCID-D: structured clinical interview for dissociative disorders

* *p*=<0.05

** *p*=<0.01

*** *p* =<0.001

### Descriptive statistics and psychophysiological reactivity in stress response groups

Stress response groups were assigned by dividing patients into four groups based on RSDI reexperiencing and dissociation scores (median split): high reexperiencing with low dissociation (R+/D−), high reexperiencing with high dissociation (R+/D+), low reexperiencing with low dissociation (R−/D−), and low reexperiencing with high dissociation (R−/D+). Descriptive statistics of distribution of diagnoses and frequencies of multiple traumatization in stress response groups are shown in Table 3. Script-related subjective reactions, symptom measures, age during first trauma and psychophysiological reactions for the groups are shown in Table 4. Between-group comparison revealed significant differences for SUD, IES total, and age during first trauma, with the highest SUD and IES scores in the high-reexperiencing (R+) groups and the lowest age during first trauma in the high-dissociation (D+) groups.

**Table 3:** Prevalence of PTSD, dissociative disorders, and multiple traumatization in stress response groups

	Stress response groups				Comparison	
		
	R+/D− (*n* =16)	R+/D+ (*n*=15)	R−/D− (*n*=15)	R−/D+ (*n*=15)	Chi-square	*p*
PTSD, *n* (%)	12 (75)	9 (60)	9 (60)	12 (80)	2.4	n.s.
Any dissociative disorder, *n* (%)	7 (44)	8 (53)	1 (7)	9 (60)	10.6	0.006
Multiple traumatizations, *n* (%)	10 (63)	13 (87)	7 (47)	13 (87)	8.4	0.36

R: script-related reexperiencing; D: script-related dissociation; +: top 50%; −: bottom 50%.

**Table 4:** Descriptive statistics of RSDI scale means, SUD, trauma-related symptoms, and psychophysiological reactivity in stress response groups

	Stress response groups				Analysis of variance (GLM)				
		
	R+/D− (*n*=16)	R+/D+ (*n*=15)	R−/D− (*n*=15)	R−/D+ (*n*=15)	Between subjects effects			Post hoc comparison R+/D− vs. R+/D+	Post hoc comparison R−/D− vs. R−/D+
Questionnaire data	Mean (95% CI)	Mean (95% CI)	Mean (95% CI)	Mean (95% CI)	F (3,57)	*p*	Eta2	*p*	*p*
RSDI reexperiencing	5.13 (4.74–5.52)	4.67 (4.26–5.07)	2.43 (2.03–2.84)	1.75 (1.35–2.15)	68.9	<0.001	0.78	Ns	0.020
RSDI avoidance	3.92 (3.03–4.80)	2.76 (1.84–3.67)	4.31 (3.40–5.23)	3.93 (3.02–4.86)	2.2	0.101	0.10	Ns	ns
RSDI dissociation	0.30 (0–0.69)	3.08 (2.67–3.49)	0.52 (0.11–0.93)	3.47 (3.06–3.87)	68.7	<0.001	0.78	<0.001	<0.001
SUD	8.25 (7.39–9.10)	7.07 (6.18–7.95)	4.93 (4.05–5.82)	4.73 (3.85–5.62)	15.2	<0.001	0.45	ns	ns
IES total	46.9 (39.7–54.1)	52.3 (45.1–59.4)	35.5 (28.4–42.7)	44.1 (36.9–51.2)	3.8	0.015	0.17	ns	ns
Age during first trauma	23.1 (16.6–29.6)	12.3 (5.6–19.0)	19.3 (12.7–26.0)	10.7 (4.0–17.4)	3.1	0.033	0.14	0.025	ns
Physiological data									
HR difference (bpm)	19.2 (14.7–23.7)	9.0 (4.3–13.6)	5.0 (0.3–9.6)	3.9 (−0.7–8.6)	9.4	<0.001	0.33	0.002	ns
HR maximum	114.8 (106–123)	96.0 (88–104)	95.9 (88–104)	90.0 (82–98)	6.9	<0.001	0.27	0.002	ns
(ln)RMSSD difference (ms)	0.40 (0.27–0.53)	0.19 (0.06–0.32)	0.07 (−0.07 to 0.20)	−0.01 (−0.14–0.13)	7.3	<0.001	0.28	0.029	ns
FPV difference (%)	−8.2 (−11.0 to −0.5.4)	−3.5 (−6.4 to −0.6)	−6.3 (−9.2 to −3.3)	−3.7 (−6.6 to −0.8)	2.4	0.075	0.11	0.025*	ns

RSDI: Responses to Script-Driven Imagery Scale; SUD: subjective units of distress; IES: Impact of Event Scale; R: script-related reexperiencing; D: script-related dissociation; +: top 50%, −: bottom 50%

* *p* value not significant after correction for influences of gender and medication

Comparison of psychophysiological reactivity during trauma script in the four stress response groups revealed significant between-group differences in HR reactivity and changes in parasympathetic cardiac activity (RMSSD). Pairwise comparison of the R+/D− and R+/D+ groups revealed significantly lower HR reactivity in the R+/D+ group (ΔHR=8.2 vs. 19.4 bpm, *p*=0.002) as well as significant higher maximum HR in the R+/D+ (HR-max=19.2 vs. 9.0 bpm, *p*=0.002). Significant between-group differences were also found for parasympathetic cardiac activity (RMSSD), with a significantly greater parasympathetic decrease in R+/D− compared to R+/D+ (Δ(ln)RMSSD=−0.38 vs. −16 ms, *p*=0.029). Supporting our main hypothesis, the statistical significance of the observed differences between R+/D− and R+/D+ in HR reactivity remained stable even when the RSDI reexperiencing score was added as a covariate (F(3,61) 2.8, *p*=0.047; pairwise comparison of groups R+/D+ vs. R+/D−: *p*=0.008). For details of the group comparisons, see Table 4.

**Fig. 1:**
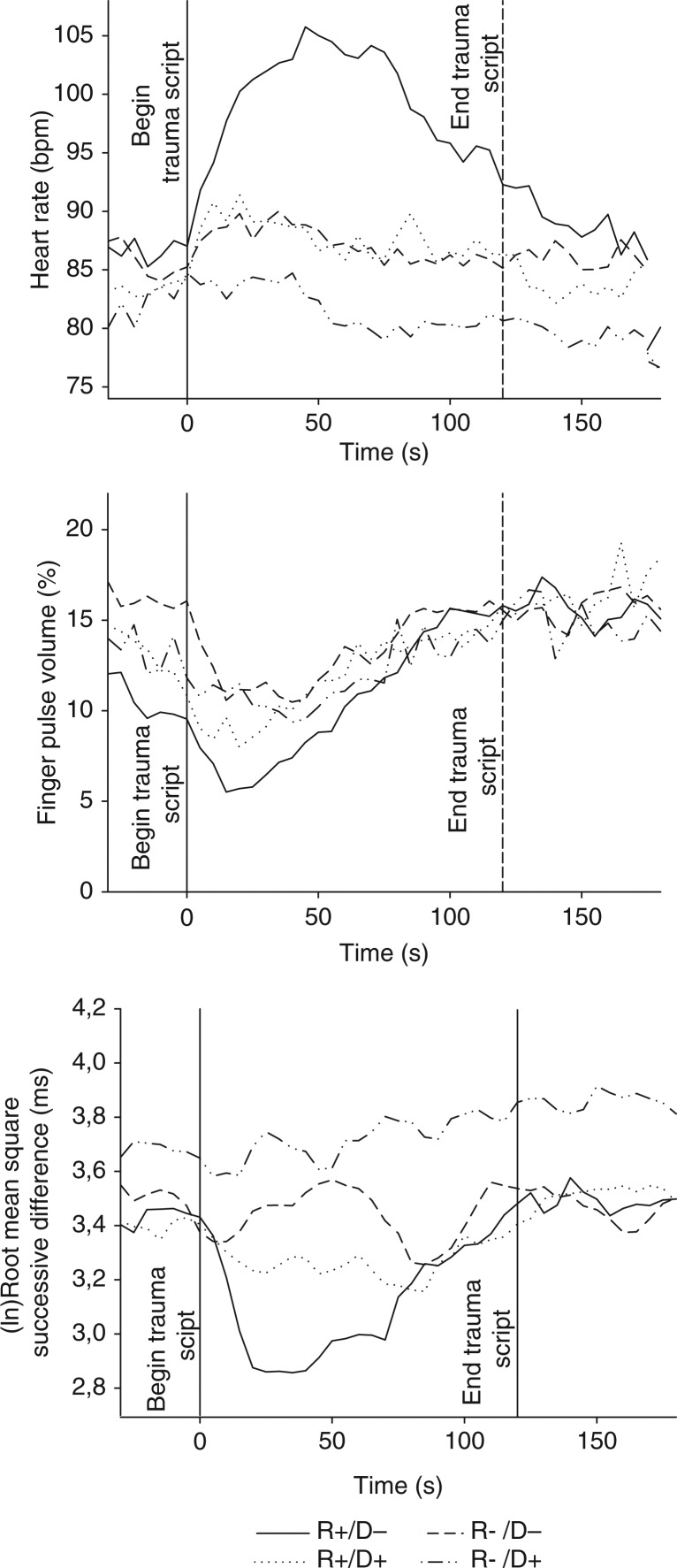
Time course of heart rate, finger pulse volume, and root mean square successive difference during trauma script in stress response groups.

To convey the time course of script-related reactions in HR, FPV and RMSSD, consecutive 5-sec mean values for each stress response group are illustrated in Fig. 1. Visual inspection of the graphs clearly reveals that HR reactions to trauma script are greater in the R+/D− group than the other three groups. The R+/D− group also has a markedly greater decrease in RMSSD during trauma script compared to the other groups. Only small group differences in FPV are apparent but, consistent with the other findings, the R+/D− group had the lowest values.

## Discussion

This study investigated psychological and psychophysiological stress responses during a reminder of the most distressing traumatic event in a sample of outpatients reporting major life trauma and a variety of trauma-associated symptoms. We found a significant association between acute dissociative symptoms and reduced psychophysiological arousal by comparing a subgroup with relatively high reexperiencing and low acute dissociative symptoms to another with high reexperiencing and high dissociation. Furthermore, the connection between acute dissociative symptoms and reduced psychophysiological arousal was reflected in negative correlations between both HR increase and HR maximum during trauma script and script-related dissociation. Participants exhibiting more severe acute dissociative symptoms during trauma script also showed a significantly diminished stress-induced decrease of parasympathetic cardiac activity in comparison to those with less severe acute dissociative symptoms. Taken together, these results confirm the main hypothesis of the study by demonstrating the association of acute dissociative stress reactions with significant inhibition of psychophysiological arousal, as reflected in reactivity of HR and parasympathetic cardiac activity.

It should be emphasized that reexperiencing clearly had a dominant effect on psychophysiological reactions during exposure to the trauma memory. Nevertheless, our results demonstrate that acute dissociation, at least at certain levels of severity, may override typical effects and inhibit psychophysiological arousal during a stressful traumatic reminder. In our study, the assessment of psychological reactions immediately after presentation of the trauma script with the RSDI (Hopper et al., 2007a), a specially designed measure of state PTSD and dissociative symptoms evoked by script-driven trauma imagery, proved effective at distinguishing reexperiencing, avoidance, dissociative dimensions, and subtypes of acute stress reactions. Interestingly, script-related avoidance symptoms did not significantly correlate with psychophysiological reactions during trauma script or with SUD, which indicates that avoidance did not successfully inhibit individual stress reactions.

The relatively dominant influence of reexperiencing over dissociation on psychophysiological reactivity may account for negative findings in several prior studies. Specifically, several studies (Kaufman et al., 2002; Ladwig et al., 2002; Nixon et al., 2005) have tested associations between psychophysiological stress reactions and retrospectively assessed peritraumatic dissociation or symptoms of trait dissociation, which are not necessarily closely related to acute dissociative stress reactions during the experiment. Since acute dissociative symptoms might contribute to the so-called “non-responding” in script-driven imagery research, we suggest that assessment of these symptoms be standard practice in future studies investigating psychophysiological stress reactions in traumatized populations.

Interestingly, our findings can be linked to findings in non-human research literature, where a distinction is made between “active” and “passive” coping reactions during an experimental stressor. While active coping is characterized by increased HR, sympathetic activation, and parasympathetic deactivation, passive coping is characterized by a parasympathetic-mediated bradycardia, often in association with signs of increased sympathetic arousal (Bosch et al., 2001; Bosch, De Geus, Veerman, Hoogstraten & Nieuw Amerongen, 2003). Passive coping reactions are often described as “conservation withdrawal” reactions, a term coined by Engel and Schmahle (1972) for a phenomenon which has much overlap with the more modern definition of acute dissociative stress responses during traumatic stress. Therefore the results of the present study suggest that the investigation of passive coping reactions might be necessary to fully understand stress reactions in traumatized populations.

Interestingly, we found a significant correlation between age during first traumatization and dissociative reactions as measured after script presentation. Correspondingly, mean age during first traumatization was significantly lower in the high-dissociation/high-reexperiencing group compared with the low-dissociation/high-reexperience group. This finding supports other research associating early life trauma with a special risk for suffering from dissociative symptoms or dissociative disorders during later life (Chu, Frey, Ganzel & Matthews, 1999; Diseth, 2006; Pasquini, Liotti, Mazzotti, Fassone & Picardi, 2002).

This study had methodological limitations. First, group assignment for statistical comparison was carried out by applying a median split on script-related reexperiencing and dissociation scores, but there was no separate control group. In purely methodological terms, it might have been preferable to induce both dissociation and reexperiencing subsequently during different experimental conditions, so that participants could serve as their own controls. However, results from previous research (Lanius et al., 2001) show that it is relatively difficult to provoke discrete reactions of either reexperiencing or dissociation, since script-driven imagery is typically associated with a range of individually different reactions that may include both dissociation and reexperiencing. Second, since the sample employed in our study was characterized by a broad spectrum of trauma-related symptoms and diagnoses, it remains open whether our results are valid also for specific diagnoses such as PTSD or dissociative disorders. Third, as it has recently been pointed out by Berntson et al. (2005), the use of RMSSD to index parasympathetic tone might have led to an underestimation of possible between-group differences in basal parasympathetic activity. For our study, RMSSD was chosen since it is especially suitable for measuring relatively short-term changes in parasympathetic activity and displaying time course data. Also, estimates of parasympathetic activity generated from frequency domain methods generally require cautious interpretation because setting factors such as breathing rate or physical activation are known to interfere significantly (Grossman, Wilhelm & Spoerle, 2004). To limit possible influences of such factors in our study, RMSSD analyses were adjusted for influences of baseline breathing rate and the experimental setting was standardized as much as possible.

The results of this study add empirical evidence to the literature on psychophysiological concomitants of dissociative reactions, by showing that acute dissociation upon exposure to trauma-related stimuli can be associated with substantial inhibition of psychophysiological arousal. While inconsistent results have been reported from studies investigating associations of psychophysiological arousal with trait dissociative symptoms or retrospectively assessing peritraumatic dissociation, the two prior studies that assessed acute dissociative stress responses immediately after a traumatic reminder each found a significant inhibition of psychophysiological arousal (Ebner-Priemer et al., 2005; Lanius et al., 2002). Psychologically traumatized individuals are at risk to experience acute dissociative symptoms during experimental paradigms that provoke psychophysiological stress reactions, and the current findings suggest that this may partly account for the so-called “non-responders” and false negative findings in the PTSD psychophysiology literature. By differentiating dissociative stress reactions from hyperarousal, our results add evidence to the conceptualization of a dissociative subtype of PTSD (Lanius et al., 2010). We therefore suggest that future research on psychophysiological stress reactions in traumatized samples should routinely assess for acute dissociative symptoms. Experimental research comparing biological concomitants of stress reactions in patients suffering from dissociative disorders with other patient groups could help to further clarify the neurobiology characteristics of dissociation.
